# *Macrophthalmus* (*Macrophthalmus*) *abbreviatus* Manning & Holthuis, 1981, a new natural host for *Hematodinium perezi* infection

**DOI:** 10.3389/fcimb.2023.1328872

**Published:** 2024-01-04

**Authors:** Zhengmin Liu, Guosi Xie, Hailiang Wang, Xinshu Li, Xiaoyuan Wan, Ang Li, Liqing Zhou, Chengyin Shi, Qingli Zhang, Jie Huang

**Affiliations:** ^1^ State Key Laboratory of Mariculture Biobreeding and Sustainable Goods, Yellow Sea Fisheries Research Institute (YSFRI), Chinese Academy of Fishery Sciences (CAFS), Qingdao, China; ^2^ Laoshan National Laboratory, Qingdao, China; ^3^ Key Laboratory of Maricultural Organism Disease Control, Ministry of Agriculture and Rural Affairs, Qingdao Key Laboratory of Mariculture Epidemiology and Biosecurity, Qingdao, Shandong, China; ^4^ School of Marine Science and Fisheries, Jiangsu Ocean University, Lianyungang, China; ^5^ Network of Aquaculture Centres in Asia-Pacific, Bangkok, Thailand

**Keywords:** parasitic dinoflagellates, *Hematodinium* sp., *Hematodinium perezi*, *Macrophthalmus abbreviatus*, host

## Abstract

Recent reports have shown that wild crabs may be important hosts involved in the transmission and spread of the parasitic *Hematodinium* in cultured marine crustaceans. Therefore, monitoring the prevalence of *Hematodinium* infections in wild crabs is necessary to develop effective strategies for the prevention and control of *Hematodinium* disease. Here we report a wild crab species, *Macrophthalmus* (*Macrophthalmus*) *abbreviatus* Manning & Holthuis, 1981, as a new natural host for *Hematodinium* sp. infection. It is one of the common wild crab species dwelling in the ponds or waterways connected to the polyculture ponds located on the coast of Rizhao or Weifang, Shandong Peninsula, China. According to the results of PCR detection and phylogenetic analysis targeting the internal transcribed spacer 1 (ITS 1) region, these *Hematodinium* sp. isolates were identified as *H. perezi* and fell into the genotype II category within *H. perezi*. A high monthly prevalence of *H. perezi* infection was observed during the 2021–2022 field survey, ranging from 33.3% to 90.6% in *M. abbreviatus* originating from Weifang (n=304 wild crabs) and from 53.6% to 92.9% in those from Rizhao (n=42 wild crabs). Artificial inoculation infection experiments demonstrated that *M. abbreviatus* could be infected by *H. perezi*, and massive *Hematodinium* cells and typical histopathological changes were observed in the hepatopancreas and gill tissues of the infected crabs. To our knowledge, this is the first report of *M. abbreviatus* as a new natural host for *H. perezi* infection. Results in the present study extend the known host spectrum for this emerging parasite pathogen, and also provide valuable information for epidemic surveillance of the *Hematodinium* disease as well.

## 1 Introduction

The parasitic dinoflagellate *Hematodinium* spp., first reported in 1931 (Chatton and Poisson, 1931), infect more than 40 crustacean species worldwide (Li et al., 2021a). This parasite has been recognized as an important pathogen causing massive mortality and extensive economic losses to some economic crustaceans (Field et al., 1992; Taylor and Khan, 1995; Messick and Shields, 2000; Shields et al., 2007; Xu et al., 2007; Li et al., 2008; Ryazanova et al., 2010; Xu et al., 2010; Albalat et al., 2012; Small, 2012; Li et al., 2013; Smith et al., 2015; Stentiford et al., 2015; Wang et al., 2017; Huang et al., 2021; Ryazanova et al., 2021). Lack of knowledge in the pathogenic and transmission mechanisms of *Hematodinium* in crustaceans has hindered the development of effective management strategies for the *Hematodinium* disease (caused by the pathogenic *Hematodinium* spp.).

To date, six wild crab species, i.e. *Helice tientsinensis*, *Uca arcuate*, *Hemigrapsus penicillatus*, *Helice wuana*, *Macrophthalmus japonicas*, and *Hemigrapsus takanoi*, inhabiting the waterways connected to the polyculture ponds of marine crustaceans, have been identified as hosts for *Hematodinium* infections (Huang et al., 2019; Li et al., 2021b; Gong et al., 2023). The prevalence of *H. perezi* in *H. tientsinensis* (up to 30.1%) is much higher than in the other five wild crab species (up to 20.0%), and is positively correlated with the prevalence of *H. perezi* in cultured *Portunus trituberculatus* (Li et al., 2021b; Gong et al., 2023). *H. perezi* can be transmitted directly from *H. tientsinensis* to *P. trituberculatus*, as evidenced by the fact that healthy *P. trituberculatus* are infected by *H. perezi*, when being cohabitated with *H. perezi* infected-*H. tientsinensis* (Huang et al., 2021). The potential role of *H. tientsinensis* is highlighted in the transmission and maintenance of *Hematodinium* in the integrative culture systems (Huang et al., 2019; Huang et al., 2021).

In the years of 2021 and 2022, we carried out epidemiological surveys for the *Hematodinium* disease in wild and cultured populations of marine crustaceans, inhabiting along the coast (mainly in Rizhao and Weifang) of the Shandong Peninsula. This area is a major mariculture region for *P. trituberculatus*, contributing to over 80% of the gross outcome in northern China (data from China Fishery Statistical Yearbook in 2021). Among the wild crab species sampled in the surveys, *M. abbreviatus* was finally identified as a novel host for *H. perezi*, based on evidences from morphological observation, molecular detection, experimental challenge tests, and histopathological examination. The prevalence of *H. perezi* in *M. abbreviatus* was also presented. The results in the present study provide important information on the *H. perezi* epidemics, and will contribute to the development of an effective prevention and management strategy for this parasite in marine crustacean pond systems.

## 2 Materials and methods

### 2.1 Crab samples and the experimental conditions

During June to October in 2021 and June to August in 2022, a total of 346 *M. abbreviatus* crabs were collected from the waterways connecting to polyculture ponds, located on the coast of Rizhao or Weifang, Shandong Province, China. These crabs were transported to the laboratory in plastic tanks with constant aeration.

In the laboratory, crabs were housed individually in perforated plastic boxes (108×108 × 42 mm, 24 holes with diameter of 3 mm) and cultured with aeration in the tanks, containing 40 L of seawater (24 ± 1°C, salinity of 30 ppt). They were fed with clams, and the residuals of clam tissue were removed timely after each feeding. 80% of culturing seawater in each tank was exchanged every day. Crabs were acclimated for a week before artificial inoculation tests.

### 2.2 Hemolymph smear assay

Hemolymph smear assays were performed as described by Stentiford and Shields (2005). Briefly, 2–3 drops of hemolymph, drawn from the juncture (prior sterilization with 70% ethanol) between the basis and ischium of the 5th walking leg, were mixed with an aliquots of neutral red (0.04%, w/v). The hemolymph smear was then screened under a light microscope (Olympus, Japan) for diagnosing and observing the status of *Hematodinium*.

### 2.3 Molecular detections for *Hematodinium*

Genomic DNA (gDNA) of hemolymph (~30 μl) sampled from natural *Hematodinium*-infected crabs and abdominal muscle (~30 mg) sampled from artificial *Hematodinium*-infected crabs were extracted by using a TIANamp Blood DNA Kit (Tiangen, China) or a TIANamp Marine Animals DNA Kit (Tiangen, China) according to the manufacturer’s instructions, respectively.

*Hematodinium* were detected using nested PCR with two primer sets as previously described by Gong et al. (2023). The nested PCR targets an interval region between 18S ribosomal RNA gene and the first internal transcribed spacer (ITS 1). The outer primer set includes primers He.p-18sF1 (CAG CTC GTG CTG ATT ACG TCC C) and He.p-ITS1R1 (AAA GCC CTA ACC CCG CTA AAG G), and the inner primer set includes primers He.p-18sF2 (TCG TAA CAA GGT TTC CGT AGG T) and He.p-ITS1R2 (ATG GAG GAG TTC AGT GGT AGG C). The PCR reaction mix (25 μL) composed of 12.5 μl Premix PrimeSTAR HS (TaKaRa, Dalian, China), 1μl exacted gDNA (~10 ng), 1 μl forward and reverse primers (10 μM, each), and 9.5 μl sterile water, respectively. PCR amplifications were conducted at 95°C for 5 min, 35 cycles (94°C for 30 s, 60°C (for outer primers)/55°C (for inner primers) for 30 s, and 72°C for 60 s (for outer primers)/20 s (for inner primers)), and 72°C for 5 min. The amplicons were analyzed using 1.2% agarose gel electrophoresis, and positive products were further sequenced with the corresponding amplification primers in Sangon Biotech (Shanghai, China) Co., Ltd.

### 2.4 Phylogenetic analysis

Eight *Hematodinium* sp. ITS1 sequences (308-341 bp in length) were retrieved from naturally *Hematodinium*-infected *M. abbreviatus* in the present study, and deposited in GenBank with Accession numbers of OQ564394 (retrieved from one crab originating from Rizhao), and OQ564393, OQ564395-OQ564400 (retrieved from seven crabs originating from Weifang). These ITS 1 sequences were multiply aligned with other *Hematodinium* sp. sequences originated from different crustacean hosts (Li et al., 2021a; Li et al., 2021b; Gong et al., 2023), using the software Clustal X. A phylogenetic tree was constructed by using the neighbor-joining method with 1000 bootstrap replicates embedded in MEGA 7.0 (Kumar et al., 2016).

### 2.5 Challenging tests

To evaluate the susceptibility of *M. abbreviatus* to *Hematodiniun* sp., artificial inoculation infection experiments were performed according to the protocol in a previous report (Li et al., 2021b). Before inoculation infections, *M. abbreviatus* were examined for *Hematodiniun* using the hemolymph smear. Negatively examined *M. abbreviatus* were used for the challenge tests. Briefly, *M. abbreviatus* (n=15) was injected with 10 µl of hemolymph, extracted from a donor *Helice tientsinensis* heavily infected by *H. perezi*, and containing 1.46 x 10^4^ parasites/µl, assessed by using a hemocytometer under a microscope. The gDNA extracted from a hemolymph sample was used for detection of *Hematodiniun* via the nested PCR assays. The PCR amplicons were confirmed after sequencing.

### 2.6 Histopathological analysis

Tissues (gills, heart, hepatopancreas, and muscle) dissected from *Hematodiniun* detection-negative or positive crabs were immediately immersed in Davidson’s AFA for 24 h. After fixation, these fixed tissues were transferred to serial alcohol and dimethylbenzene, and embedded in paraffin. The sections were stained with hematoxylin and eosin (H&E), using routine histological method as described by (Wheeler et al., 2007), and subsequently photographed under a light microscope (Olympus, Japan).

## 3 Results

### 3.1 Morphology of *Hematodinium* sp. in the hemolymph from naturally infected *M. abbreviatus*

**Figure 1:**
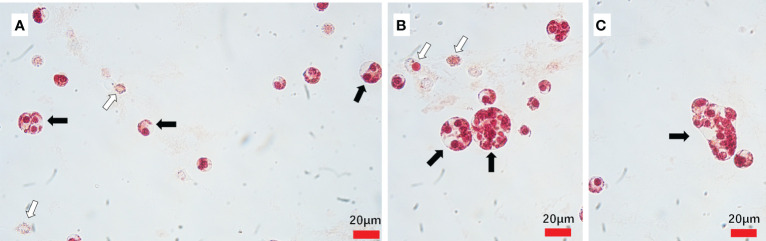
*Hematodinium* staining and examination in the hemolymph from naturally infected *M. abbreviatus*. Uninucleate, binuclear and trinuclear ameboid trophonts **(A)** Multinucleate ameboid trophonts **(B)** Putative clump colony **(C)**. Black arrows indicate parasites and white arrow indicate host haemocytes.

*Hematodinium* sp. exhibited various shapes were observed in the hemolymph from the naturally *Hematodinium*-infected crabs, which indicates different developmental stages in the lifecycle of *Hematodinium*. They were ameboid trophonts (e.g., uninucleate, binuclear, trinuclear and multinucleate) (**Figures 1A, B**) and presumptive clump colonies (**Figure 1C**).

### 3.2 Prevalence of *Hematodinium* infections in *M. abbreviatus*

**Figure 2:**
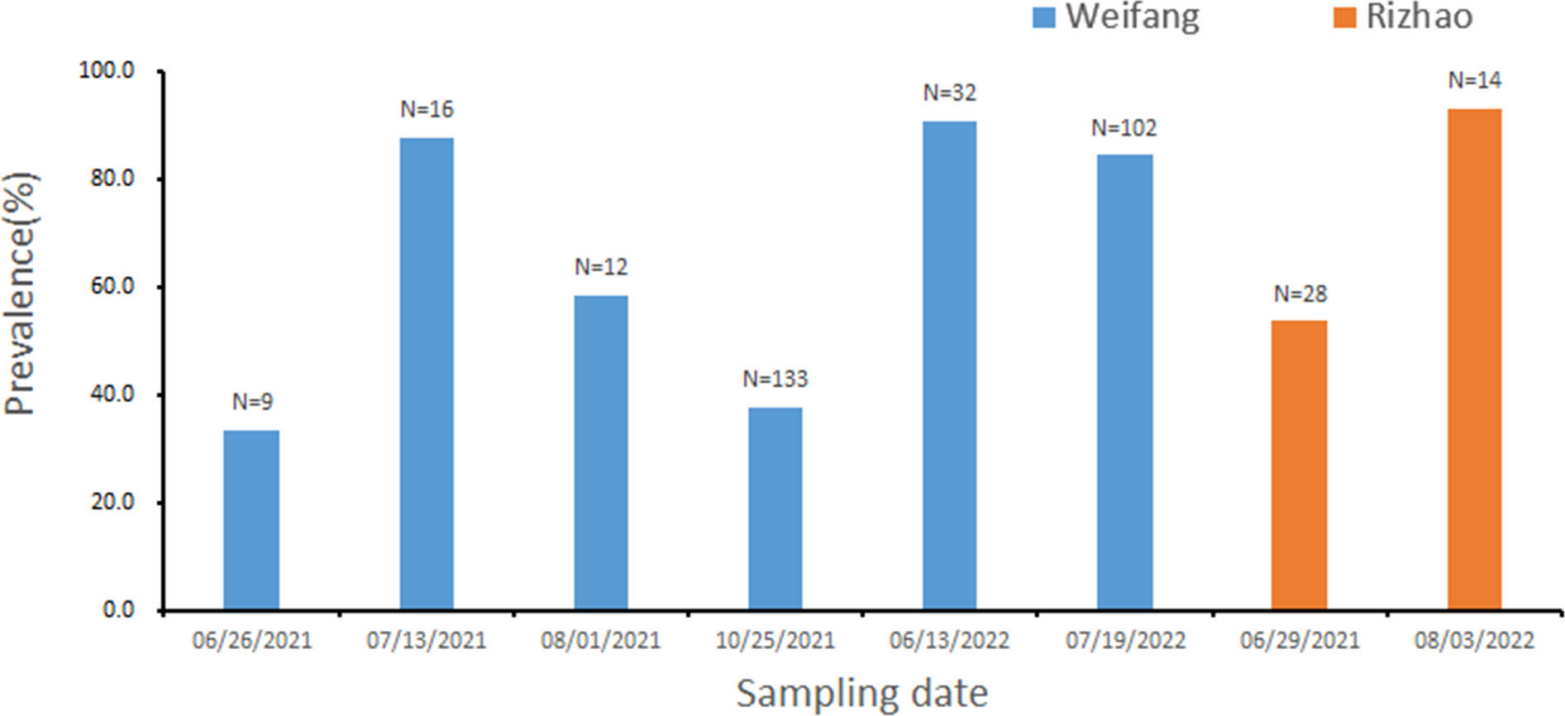
Prevalence of *Hematodinium* infections in *M. abbreviatus*, collected from Rizhao and Weifang during the sampling period. The total sample number are shown over each bar.

Considerable prevalence of *H. perezi* infections existed in *M. abbreviatus* originated from Weifang (the averaged prevalence rate of 62.2%; n=304) or Rizhao (66.7%; n=42). On the month scale, the prevalence in specific varied from 33.3% to 90.6% in Weifang samples, and from 53.6% to 92.9% in Rizhao samples (**Figure 2**). The overall prevalence in these samples originated from the two regions during investigations in years 2021 and 2022 was 62.7% (n=346).

### 3.3 Phylogenetic and taxonomic analyses

Eight *Hematodinium* ITS 1 sequences (265 or 266 bp in length) were retrieved from *M. abbreviatus* collected from Weifang and Rizhao and they shared 99.6–100% sequence similarity with each other. In addition, multiple sequence alignments showed that they shared high similarity (97.8–100%) with those of *H. perezi* genotypes I and II, which infect crustacean species such as *P. trituberculatus*, *S. paramamosain*, *P. monodon*, and *H. tientsinensis* in China (Small et al., 2012; Wang et al., 2017; Huang et al., 2019; Li et al., 2021b), while they shared relatively lower similarity (95.9%-96.7%) with that of *H. perezi* genotype III isolates, infecting *Callinectes sapidus* in USA (Jensen et al., 2010), and other *Hematodinium* isolates (≤63.5%).

**Figure 3:**
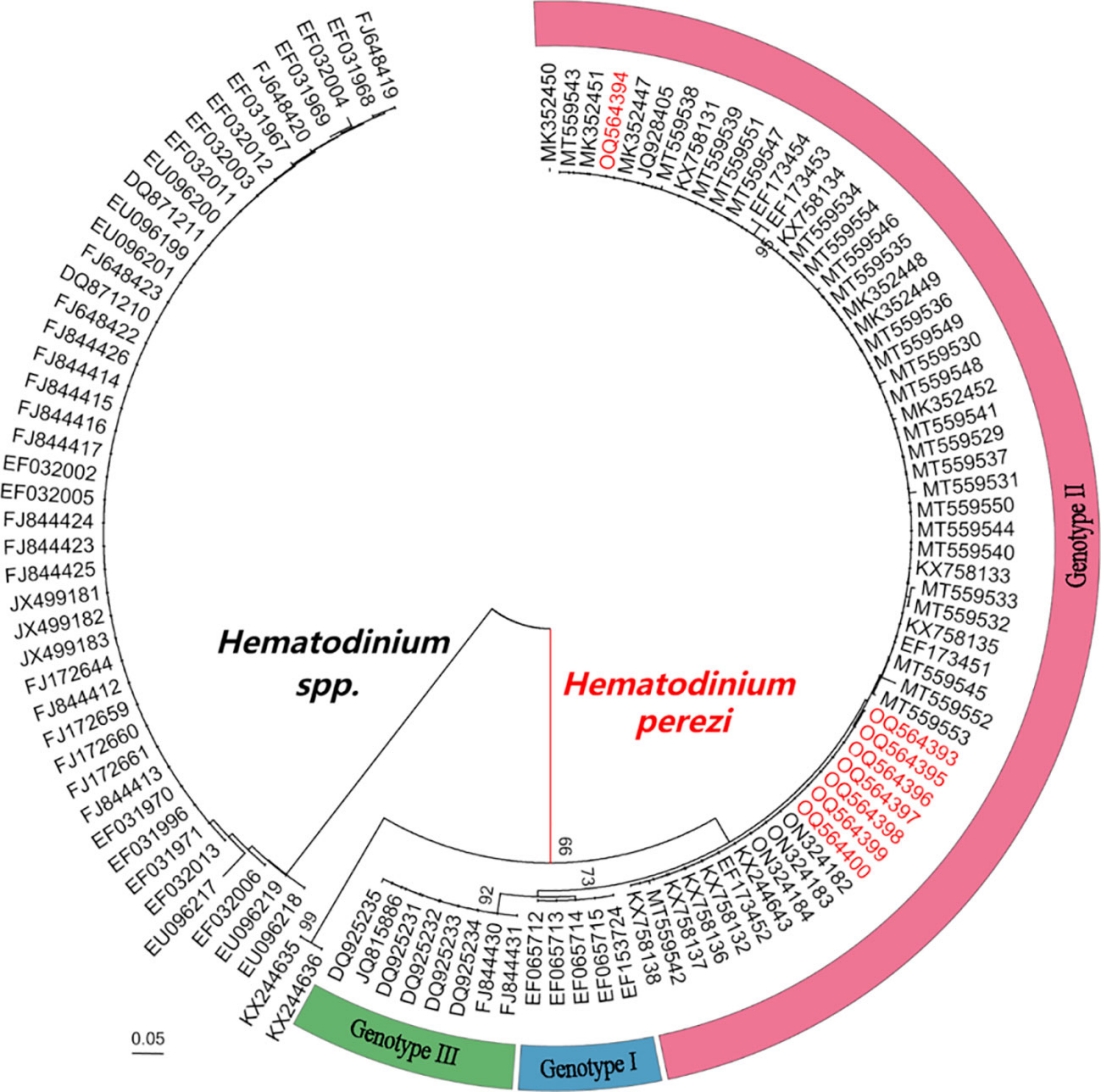
Neighbour-joining phylogenetic tree based on partial sequences of ITS 1 gene from *Hematodinium* spp. *Hematodinium* sequences were from previous reports (Li et al., 2021a; Li et al., 2021b; Gong et al., 2023). Bootstrap values above 60% are shown (1000 replications) at branch points. Bar = 0.05.

The phylogenetic analysis based on ITS1 sequences indicated that the eight *Hematodinium* isolates in the present study were all grouped into *H. perezi* genotype II (**Figure 3**), which together with that other isolates reported in China, e.g., *P. trituberculatus*, *P. monodon*, *S. paramamosain*, *H. tientsinensisn*, and *H. takanoi* (Small et al., 2012; Wang et al., 2017; Huang et al., 2019; Li et al., 2021b; Gong et al., 2023). Taken together, the *Hematodinium* isolates in *M. abbreviatus* were taxonomically identified as *H. perezi* and belonged to the genotype II.

### 3.4 *H. perezi* confirmed in the hemolymph from artificially challenged *M. abbreviatus*

**Figure 4:**
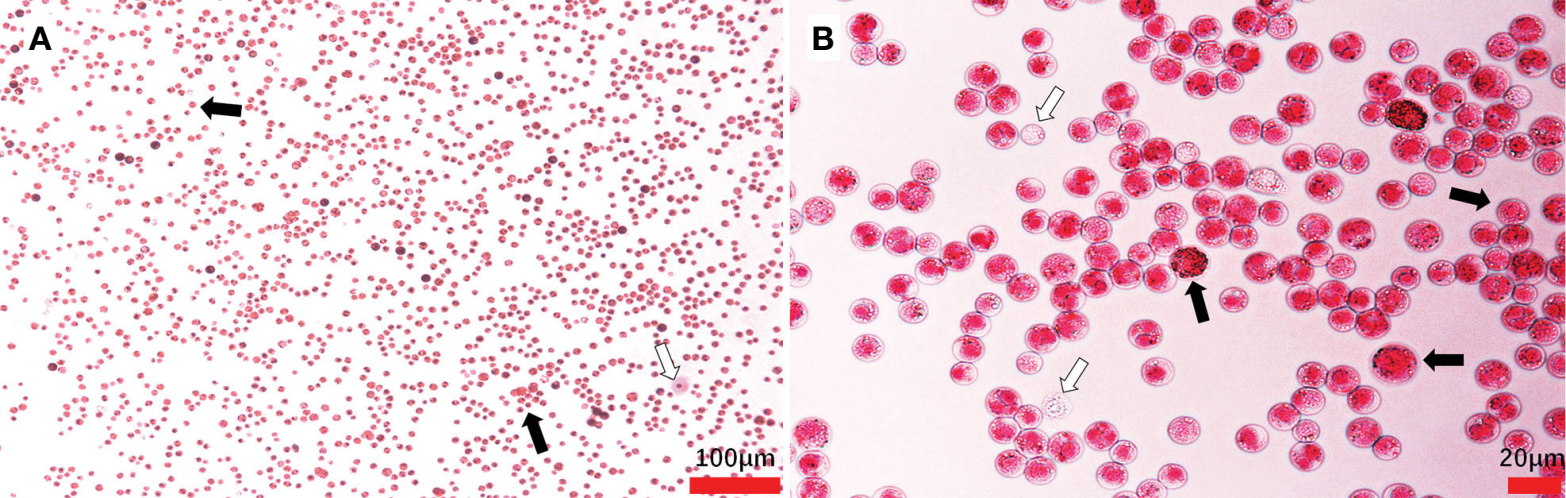
*Hematodinium* staining and examination in the hemolymph from artificially challenged *M. abbreviatus*. Black arrows indicate parasites and white arrows indicate haemocytes **(A, B)**.

Massive *Hematodinium* ameboid cells were observed in the hemolymph of artificially inoculated *H. perezi*-infected crabs, whereas haemocytes were rare in this sample (**Figures 4A, B**). The hemolymph samples were positive for *Hematodinium* and identified as *H. perezi* based on the results of PCR and phylogenetic analysis.

### 3.5 Histopathology caused by *Hematodinium* infections

**Figure 5:**
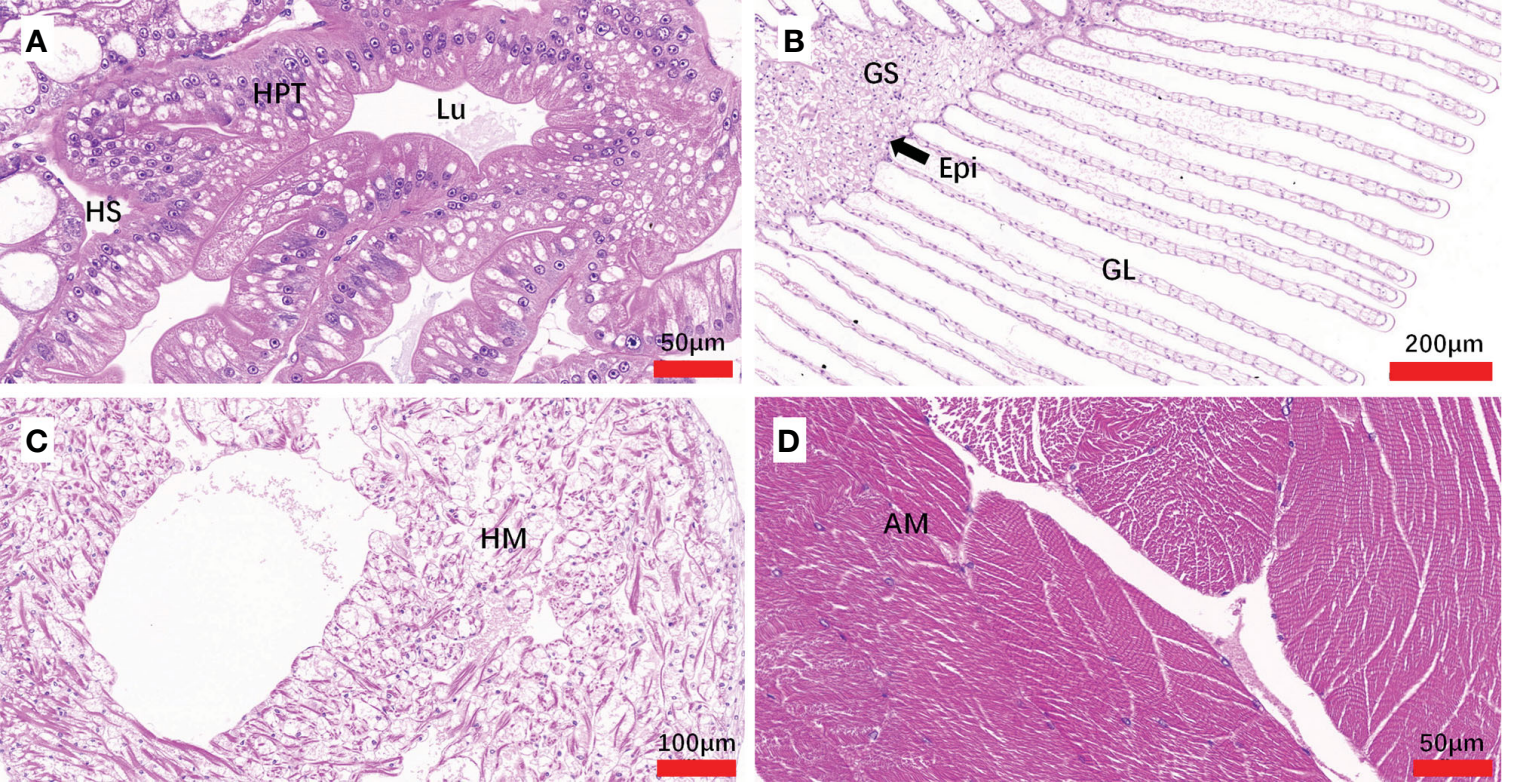
Sections of tissues from *Hematodiniun* infection-negative *M. abbreviatus.* Hepatopancrea **(A)**, gill **(B)**, heart **(C)** and abdominal muscle **(D)** tissus. H&E staining. HPT, Hepatopancreatic tubule; Lu, lumen of hepatopancreatic tubules; HS, hemal space; Epi, epithelium; GS, Gill stem; GL Gill lamellae; HM, heart muscle; AM, abdominal muscle.

**Figure 6:**
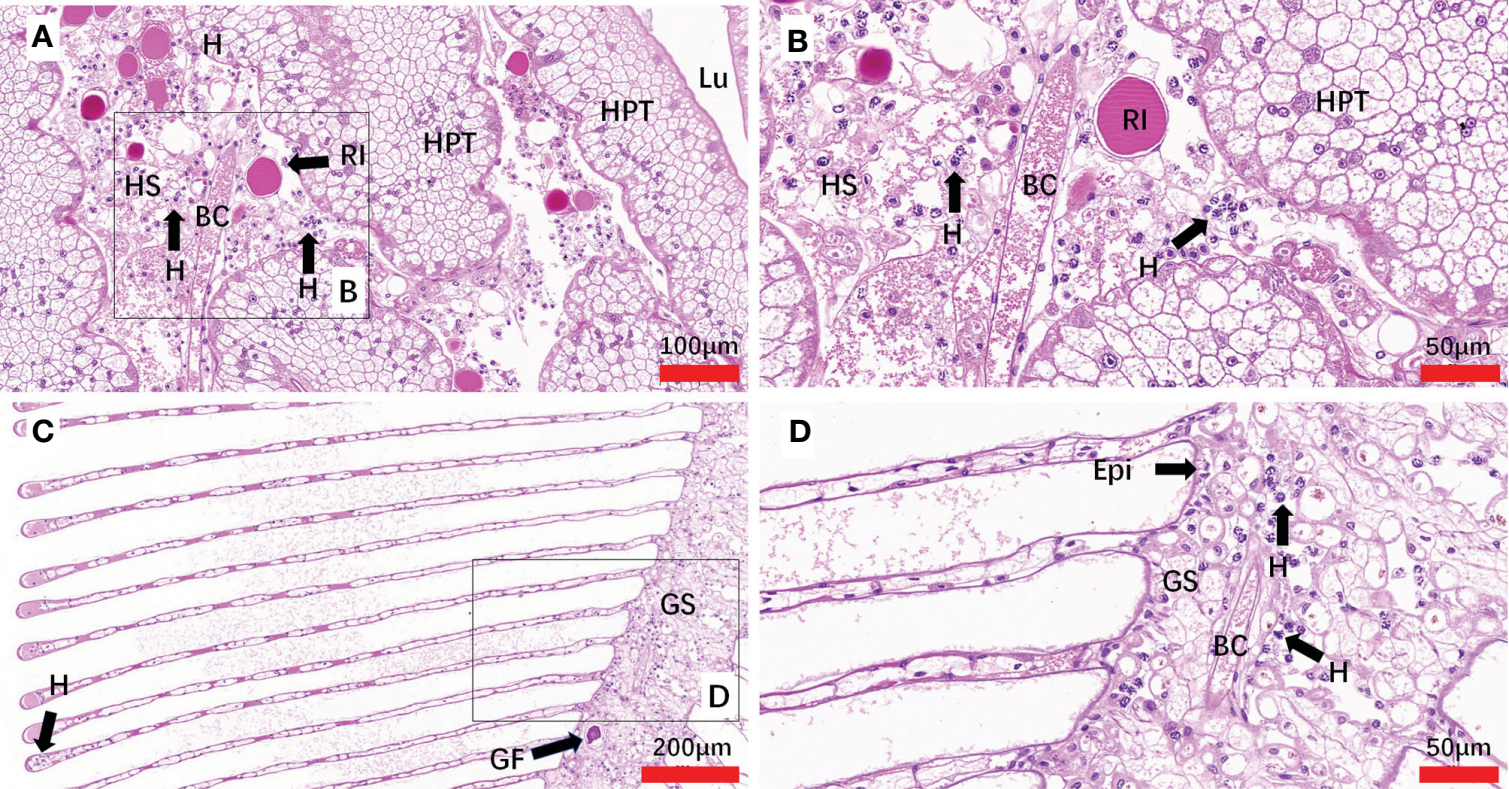
Sections of tissues from naturally *Hematodinium*-infected *M. abbreviatus.* Hepatopancrea **(A, B)** and gill **(C, D)**. Note uninucleate ameboid trophonts with V-shaped chromatin in the nucleus were observed in Hepatopancrea **(B)** and gill **(D)**. A granulomas was present in the gill tissues **(C)**. H&E staining. HPT, Hepatopancreatic tubule; Lu, lumen of hepatopancreatic tubules; HS, hemal space; BC, blood channel; H, *Hematodinium* cells; P, fixed phagocytes; RI, reserve inclusion cells; GF, granuloma formation; Epi, epithelium; GS, Gill stem; GL Gill lamellae.

The histopathology caused by natural and artificial *H. perezi* infections in *M. abbreviatus* was examined. Compared to the histology of *Hematodinium*-negative crabs (**Figures 5A–D**), obvious histopathological changes were observed in the tissues of hepatopancreas and gills of *M. abbreviatus* suffering from natural *Hematodinium* infection (**Figures 6A–D**). The prominent V-shaped chromosomes characteristic of syndinean dinoagellates were observed in hepatopancreas (**Figure 6B**) and gills (**Figure 6D**), whereas these parasites were rarely observed in heart and muscle, probably due to light infections. Granulomas were also observed in the diseased gills (**Figure 6C**).

**Figure 7:**
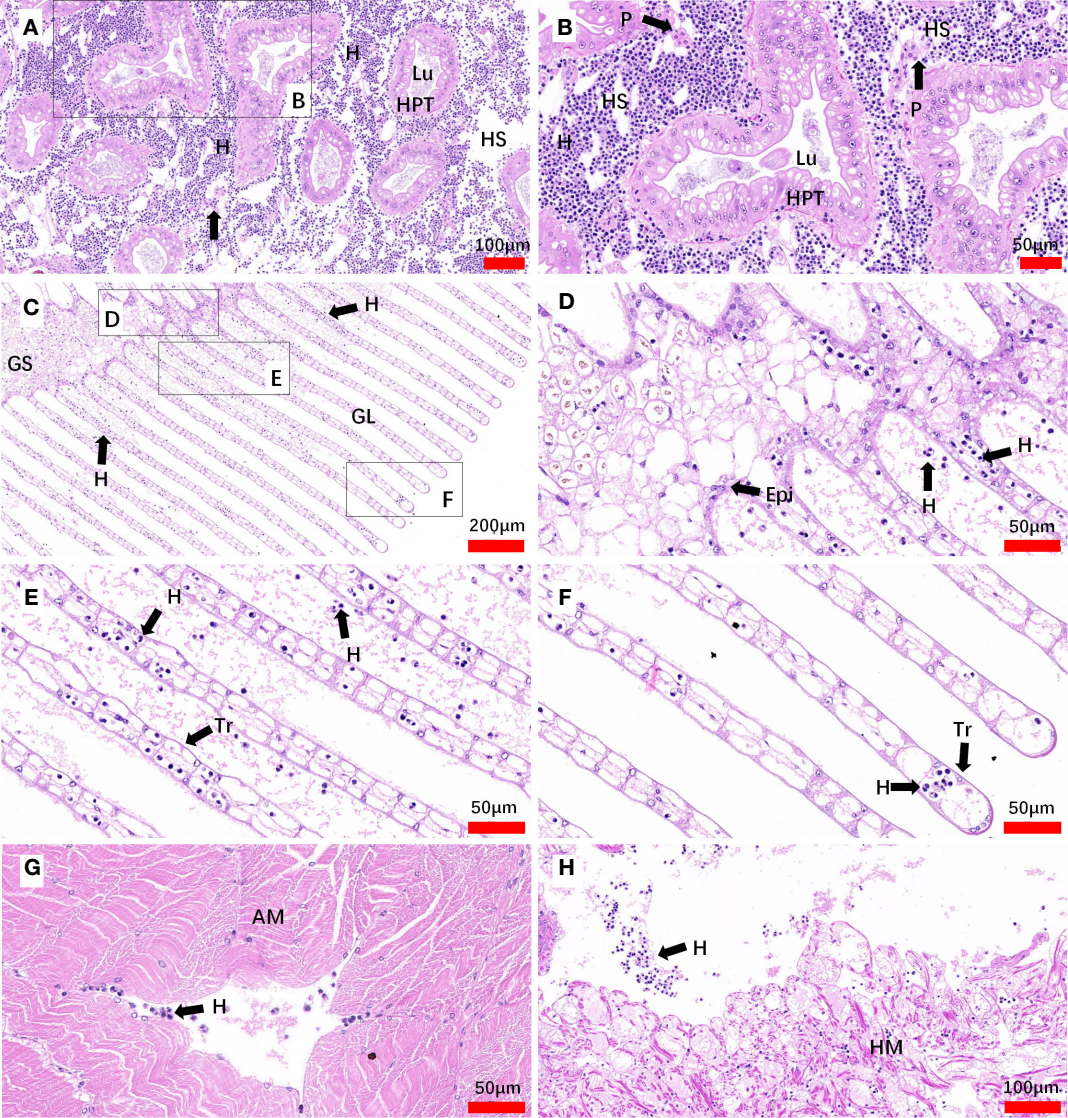
Sections of tissues from artificial inoculation *Hematodinium*-infected *M. abbreviatus.* Hepatopancrea **(A, B)**, gill **(C-F)**, abdominal muscle **(G)** and heart **(H)** tissus of infected crab. H&E staining. HPT, Hepatopancreatic tubule; Lu, lumen of hepatopancreatic tubules; HS, hemal space; H, *Hematodinium* cells; P, fixed phagocytes; Epi, epithelium; GS, Gill stem; GL Gill lamellae; Tr, trabecular cell; AM, abdominal muscle; HM, heart muscle.

In inoculated crabs at 25-day post-infection, the hepatopancreas was severely altered due to the invasion of *Hematodinium* parasites, the spongy connective tissue around the hepatopancreatic tubules appeared to be diminished, and haemocytes were barely observed. The enlarged hemal space among the hepatopancreatic tubules was filled with massive numbers of parasites, while the hepatopancreatic tubules appeared intact and no parasites were observed within the lumen proper of the hepatopancreatic tubules. In addition, fixed phagocytes which were enlarged and activated were observed, probably in response of tissue degradation or parasite infection (**Figures 7A, B**). In the gills, a number of *Hematodinium* parasites were observed in the gill stem and filaments. The epithelium was deformed and appeared to be necrotic, and reduction or loss of spongy connective tissue was also observed. In some of the gill lamellae, necrosis of the connective tissue, and loss or necrosis of the trabecular cells were observed due to parasite infection (**Figures 7C–F**). The released spores were also observed between adjacent lamellae (**Figure 7D**). In abdominal muscle tissue, the intact structure of the muscle fibers was distorted and lost its normal dense appearance due to interstitial infiltrates of parasites (**Figure 7G**). In the heart tissue, the heart muscle lost its normal dense appearance and the myocardial fibers showed a reduction or loss of spongy connective tissue, separated by parasite cell invasion (**Figure 7H**).

## 4 Discussion

Since 2013, frequent outbreaks of the *Hematodinium* disease have led to serious economic losses to the cultured *P. trituberculatus* in coastal areas of the Shandong Peninsula. Considering the fact that hosts play an important role in occurrence and spread of parasitic diseases, it is important to conduct epidemic surveillance for hosts of *Hematodinium* sp. In the present study, we demonstrated that *M. abbreviatus* is a new natural host of *H. perezi*. This finding together with previous publications show that *H. perezi* genotype II can infect various marine crustaceans (e.g. *H. takanoi*, *P. trituberculatus*, *H. tientsinensis*, *S. paramamosain* and *P. monodon*) under a wide host range in China (Small et al., 2012; Wang et al., 2017; Huang et al., 2019; Li et al., 2021b; Gong et al., 2023). The occurrence and transmission mechanisms of *Hematodinium* parasites in the integrative culture systems are not yet clearly understood. Dinospores may play an important role in the transmission of this disease. A recent study on the transmission pattern indicated that *H. perezi* can be transmitted from *H. tientsinensis* to *P. trituberculatus* via waterborne transmission, while *P. trituberculatus* could not be infected by *H. perezi* via feeding tissues from *H. perezi*-infected *H. tientsinensis* (Li et al., 2021b).

It is noted that a similar trend in the prevalence of *H. perezi* infection was observed in both *M. abbreviatus* and *P. trituberculatus* collected simultaneously during the survey in Weifang in the present study. According to the results of PCR detection, the monthly prevalence of *H. perezi* infection in *M. abbreviatus* was generally higher than that in the cultured *P. trituberculatus* (unpublished data) in 2021. When compared with the data from a previous survey from June to October in 2018 in Qingdao, China (Li et al., 2021b), which is adjacent to the sampling site in the present study, the monthly prevalence of *H. perezi* in *M. abbreviatus* was also much higher than that in *H. tientsinensis* (9.4%-30.1%) and the cultured *P. trituberculatus* (3.0%-55.7%). In addition, *M. abbreviatus* is a migratory species in the integrative culture ponds and a dominant species of the most common wild crab species, dwelling in the polyculture ponds or in the waterways connected to the polyculture pond system along the coast of the Shandong Peninsula. Therefore, it is reasonable to assume that *M. abbreviatus* as well as other co-inhabiting crabs are likely important alternates or reservoir hosts involved in transmission and maintenance of *Hematodinium*. It requires further investigations to determine whether *M. abbreviatus* is more susceptible to *Hematodinium* infection compared to *P. trituberculatus* and/or other reported wild crabs, and the exact role of *M. abbreviatus* in the epizootiology of *Hematodinium* disease.

## 5 Conclusions

In summary, to our knowledge, this is the first report of naturally infected *M. abbreviatus* with *H. perezi. M. abbreviatus* with a high prevalence of *H. perezi* infections indicates that more attention should be paid to their potential risk in *Hematodinium* epizootics. The present study extends the known hosts for *H*. *perezi*, and also provides important information for monitoring and designing effective management strategies for *Hematodinium* disease in marine crustacean aquaculture in China.

## Data availability statement

The datasets presented in this study can be found in online repositories. The names of the repository/repositories and accession number(s) can be found in the article/supplementary material.

## Ethics statement

The animal study was approved by Since the Ethical Principles and Guidelines for the Use of Animals of the National Research Council of China apply to vertebrates only, there is no official standard for invertebrates. We adapted its principles to shrimp. All of the animal experiments were performed according to local government regulations and the protocols were approved by the Animal Ethics Committee of the Yellow Sea Fisheries Institute, Chinese Academy of Fishery Sciences. The study was conducted in accordance with the local legislation and institutional requirements.

## Author contributions

ZL: Investigation, Project administration, Writing – original draft, Formal analysis. GX: Writing – original draft, Writing – review & editing, Conceptualization, Data curation, Formal analysis, Funding acquisition, Investigation, Methodology, Project administration, Resources, Software, Supervision, Validation, Visualization. HW: Formal analysis, Methodology, Supervision, Writing – original draft, Writing – review & editing. XL: Methodology, Writing – review & editing, Formal analysis. XW: Methodology, Software, Supervision, Writing – review & editing. AL: Methodology, Writing – review & editing. LZ: Methodology, Writing – review & editing. CS: Supervision, Writing – review & editing. QZ: Formal analysis, Writing – review & editing. JH: Conceptualization, Funding acquisition, Project administration, Resources, Supervision, Writing – review & editing.

## References

[B1] Albalat A. Gornik S. G. Beevers N. Atkinson R. J. A. Miskin D. Neil D. M. (2012). *Hematodinium* sp. infection in Norway lobster *Nephrops norvegicus* and its effects on meat quality. Dis. Aquat. Organ. 100, 105–112. doi: 10.3354/dao02500 23186698

[B2] Chatton E. P. L. Poisson R. (1931). Sur l'existence dans la sang des crabes, de péridiniens parasites: *Hematodinium perezi* n.g., n. sp. (Syndinidae). C.R. Seances Soc. Biol. Paris 1931 105, 553–557.

[B3] Field R. Chapman C. Taylor A. Neil D. Vickerman K. (1992). Infection of the Norway lobster Nephrops norvegicus by a *Hematodinium*-like species of dinoflagellate on the west coast of Scotland. Dis. Aquat. Organ 13, 1–15. doi: 10.3354/dao013001

[B4] Gong M. Xie G. Wang H. Li X. Li A. Wan X.. (2023). *Hematodinium perezi* naturally infects Asian brush-clawed crab (*Hemigrapsus takanoi*). J.Fish.Dis. 46, 67–74. doi: 10.1111/jfd.13718 36169647

[B5] Huang Q. Li M. Wang F. Li C. (2019). The parasitic dinoflagellate *Hematodinium perezi* infecting mudflat crabs, *Helice tientsinensis*, in polyculture system in China. J. Invertebr. Pathol. 166, 107229. doi: 10.1016/j.jip.2019.107229 31394065

[B6] Huang Q. Li M. Wang F. Song S. Li C. (2021). Transmission pattern of the parasitic dinoflagellate *Hematodinium perezi* in polyculture ponds of coastal China. Aquaculture 538, 736549. doi: 10.1016/j.aquaculture.2021.736549

[B7] Jensen P. C. Califf K. Lowe V. Hauser L. Morado J. F. (2010). Molecular detection of Hematodinium sp. in Northeast Pacific *Chionoecetes* spp. and evidence of two species in the Northern Hemisphere. Dis. Aquat. Organ. 89, 155–166. doi: 10.3354/dao02193 20402233

[B8] Kumar S. Stecher G. Tamura K. (2016). MEGA7: molecular evolutionary genetics analysis version 7.0 for bigger datasets. Mol. Biol. Evol. 33, 1870–1874. doi: 10.1093/molbev/msw054 27004904 PMC8210823

[B9] Li M. Huang Q. Lv X. Song S. Li C. (2021b). The parasitic dinoflagellate *Hematodinium* infects multiple crustaceans in the polyculture systems of Shandong Province, China. J. Invertebr. Pathol. 178, 107523. doi: 10.1016/j.jip.2020.107523 33358749

[B10] Li C. Li M. Huang Q. (2021a). The parasitic dinoflagellate *Hematodinium* infects marine crustaceans. Mar. Life. Sci. Technol. 3, 313–325. doi: 10.1007/s42995-020-00061-z 37073297 PMC10077234

[B11] Li C. Song S. Liu Y. Chen T. (2013). *Hematodinium* infections in cultured Chinese swimming crab, *Portunus trituberculatus*, in northern China. Aquaculture 396, 59–65. doi: 10.1016/j.aquaculture.2013.02.022

[B12] Li Y. Y. Xia X. A. Wu Q. Y. Liu W. H. Lin Y. S. (2008). Infection with Hematodinium sp. in mud crabs *Scylla serrata* cultured in low salinity water in southern China. Dis. Aquat. Organ. 82, 145–150. doi: 10.3354/dao01988 19149377

[B13] Messick G. A. Shields J. D. (2000). Epizootiology of the parasitic dinoflagellate Hematodinium sp. in the American blue crab *Callinectes sapidus* . Dis. Aquat. Organ. 43, 139–152. doi: 10.3354/dao043139 11145454

[B14] Ryazanova T. Eliseikina M. Kukhlevsky A. (2021). First detection of Hematodinium sp. In spiny king crab *Paralithodes brevipes*, and new geographic areas for the parasite in tanner crab *Chionoecetes bairdi*, and red king crab *Paralithodes camtschaticus* . J. Invertebr. Pathol. 184, 107651. doi: 10.1016/j.jip.2021.107651 34348127

[B15] Ryazanova T. Eliseikina M. Kukhlevsky A. Kharlamenko V. (2010). *Hematodinium* sp. infection of red *Paralithodes camtschaticus* and blue *Paralithodes platypus* king crabs from the Sea of Okhotsk, Russia. J. Invertebr. Pathol. 105, 329–334. doi: 10.1016/j.jip.2010.07.009 20691697

[B16] Shields J. D. Taylor D. M. O’Keefe P. G. Colbourne E. Hynick E. (2007). Epidemiological determinants in outbreaks of bitter crab disease (*Hematodinium* sp.) in snow crabs *Chionoecetes opilio* from Conception Bay, Newfoundland, Canada. Dis. Aquat. Organ. 77, 61–72. doi: 10.3354/dao01825 17933398

[B17] Small H. J. (2012). Advances in our understanding of the global diversity and distribution of Hematodinium spp.–Significant pathogens of commercially exploited crustaceans. J. Invertebr. Pathol. 110, 234–246. doi: 10.1016/j.jip.2012.03.012 22433998

[B18] Small H. J. Shields J. D. Reece K. S. Bateman K. Stentiford G. D. (2012). Morphological and molecular characterization of *Hematodinium perezi* (Dinophyceae: Syndiniales), a dinoflagellate parasite of the harbour crab, Liocarcinus depurator. J. Eukaryot. Microbiol. 59, 54–66. doi: 10.1111/j.1550-7408.2011.00592.x 22092696

[B19] Smith A. L. Hirschle L. Vogan C. L. Rowley A. F. (2015). Parasitization of juvenile edible crabs (*Cancer pagurus*) by the dinoflagellate, Hematodinium sp.: pathobiology, seasonality and its potential effects on commercial fisheries. Parasitology. 142, 428–438. doi: 10.1017/S0031182014001255 25118672

[B20] Stentiford G. Neil D. Albalat A. Milligan R. Bailey N. (2015). The effect of parasitic infection by Hematodinium sp. on escape swimming and subsequent recovery in the Norway lobster, *Nephrops norvegicus* (L.). J. Crustacean. Biol. 35, 1–10. doi: 10.1163/1937240X-00002296

[B21] Stentiford G. D. Shields J. D. (2005). A review of the parasitic dinoflagellates *Hematodinium* species and *Hematodinium*-like infections in marine crustaceans. Dis. Aquat. Organ. 66, 47–70. doi: 10.3354/dao066047 16175968

[B22] Taylor D. Khan R. (1995). Observations on the occurrence of Hematodinium sp.(Dinoflagellata: Syndinidae), the causative agent of bitter crab disease in Newfoundland snow crab (*Chionoecetes opilio*). J. Invertebr. Pathol. 65, 283–288. doi: 10.1006/jipa.1995.1043

[B23] Wang J. Li M. Xiao J. Xu W. Li C. (2017). *Hematodinium* spp. infections in wild and cultured populations of marine crustaceans along the coast of China. Dis. Aquat. Organ. 124, 181–191. doi: 10.3354/dao03119 28492174

[B24] Wheeler K. Shields J. D. Taylor D. M. (2007). Pathology of *Hematodinium* infections in snow crabs (*Chionoecetes opilio*) from Newfoundland, Canada. J. Invertebr. Pathol. 95, 93–100. doi: 10.1016/j.jip.2007.01.002 17336326

[B25] Xu W. Shi H. Xu H. Small H. (2007). Preliminary study on the *Hematodinium* infection in cultured *Portunus trituberculatus* . Acta Hydrobiologica Sin. 31, 637–640. doi: 10.3321/j.issn:1000-3207.2007.05.004

[B26] Xu W. Xie J. Shi H. Li C. (2010). *Hematodinium* infections in cultured ridgetail white prawns, *Exopalaemon carinicauda*, in eastern China. Aquaculture 300, 25–31. doi: 10.1016/j.aquaculture.2009.12.024

